# Gastrointestinal Basidiobolomycosis in a 45-Year-Old Woman

**DOI:** 10.7759/cureus.12109

**Published:** 2020-12-16

**Authors:** Lulwah Alabdan, Sadiq M Amer, Zainab Alnabi, Noor Alhaddab, Sami Almustanyir

**Affiliations:** 1 Internal Medicine, Prince Mohammed Bin Abdulaziz Hospital, Riyadh, SAU; 2 Pathology, Prince Mohammed Bin Abdulaziz Hospital, Riyadh, SAU; 3 College of Medicine, Alfaisal University, Riyadh, SAU; 4 Internal Medicine, Ministry of Health, Riyadh, SAU

**Keywords:** basidiobolomycosis, basidiobolus ranarum, splendore–hoeppli phenomenon, gastrointestinal

## Abstract

Basidiobolomycosis is an infrequent fungal infection. It is largely a subcutaneous infection and its gastrointestinal involvement is an uncommon phenomenon. Herein, we report the case of gastrointestinal basidiobolomycosis in a 45-year-old Saudi woman who presented to the clinic with a three-week history of abdominal pain. Although infrequent, however, gastrointestinal basidiobolomycosis should be contemplated in patients presenting with abdominal pain, hematologic eosinophilia, and inflammatory gastrointestinal mass.

## Introduction

Basidiobolomycosis is an infrequent fungal infection [1]. It is most often caused by *Basidiobolus ranarum*, a saprophytic fungus that is affiliated with the Zygomycetes family [1]. Skin and subcutaneous tissues are the most typically implicated sites of infection [2,3]. Nonetheless, the involvement of the gastrointestinal tract by basidiobolomycosis is exceedingly rare [4,5]. It is approximated that less than 135 cases of gastrointestinal basidiobolomycosis have been recorded so far in the literature [1,4,5]. The vast majority of the published cases were in males and children [1,4,5]. Herein, we report the case of gastrointestinal basidiobolomycosis in a 45-year-old Saudi woman who presented to clinical attention with a five-day history of abdominal pain.

## Case presentation

A 45-year-old Saudi woman presented to the emergency department complaining of abdominal pain for five days. The pain was diffuse, aching, eight of ten for severity, and non-radiating. It was associated with nausea, vomiting, and constipation, but no blood in the stool. Past medical history was remarkable for diabetes mellitus and hypertension. Past surgical history was notable for appendectomy nine years ago.

On physical examination, vital signs were remarkable for fever of 37.9°C (normal range: 36.5-37.5°C) and increased respiratory rate of 24 breaths/min (normal range: less than 20 breaths/min). Abdominal examination revealed diffuse tenderness, mostly involving the right side. No palpable masses were detected. Bowel sounds were normal. A digital examination was not performed.

Initial Blood tests revealed an elevated white blood cell count of 14.9 x 10^9^/L (normal range: 4.5-12 x 10^9^/L) with a high eosinophil count of 290 x 10^3^ cells/uL (normal range: 35-400 cells/uL). Additionally, the patient had an elevated erythrocyte sedimentation rate of 112 mm/h (normal range: 0-17 mm/h). Tumor markers, including carcinoembryonic antigen (CEA) and cancer antigen 125 (CA-125), were within normal limits.

**Figure 1:**
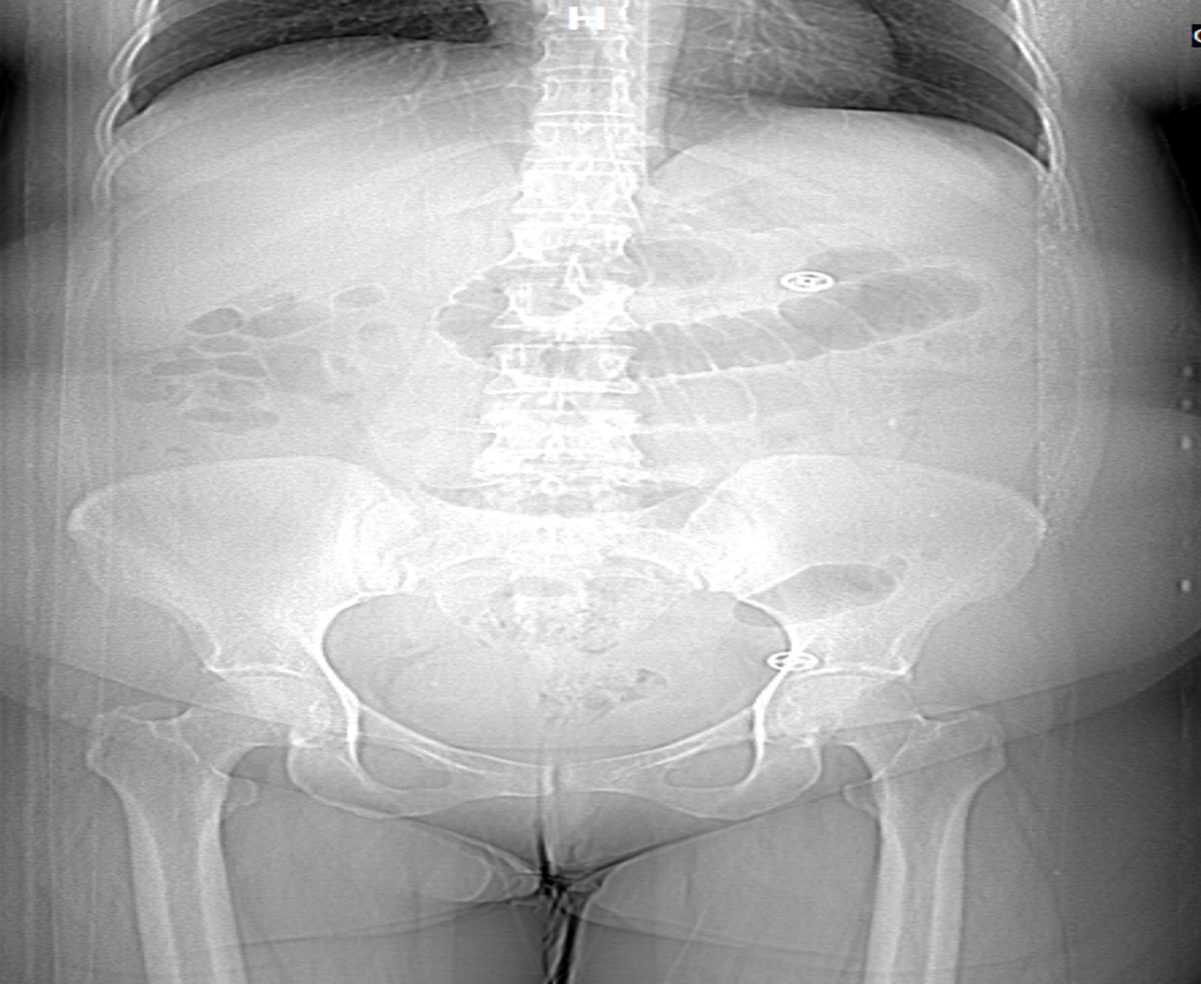
Abdominal x-ray showing multiple air-fluid levels.

**Figure 2:**
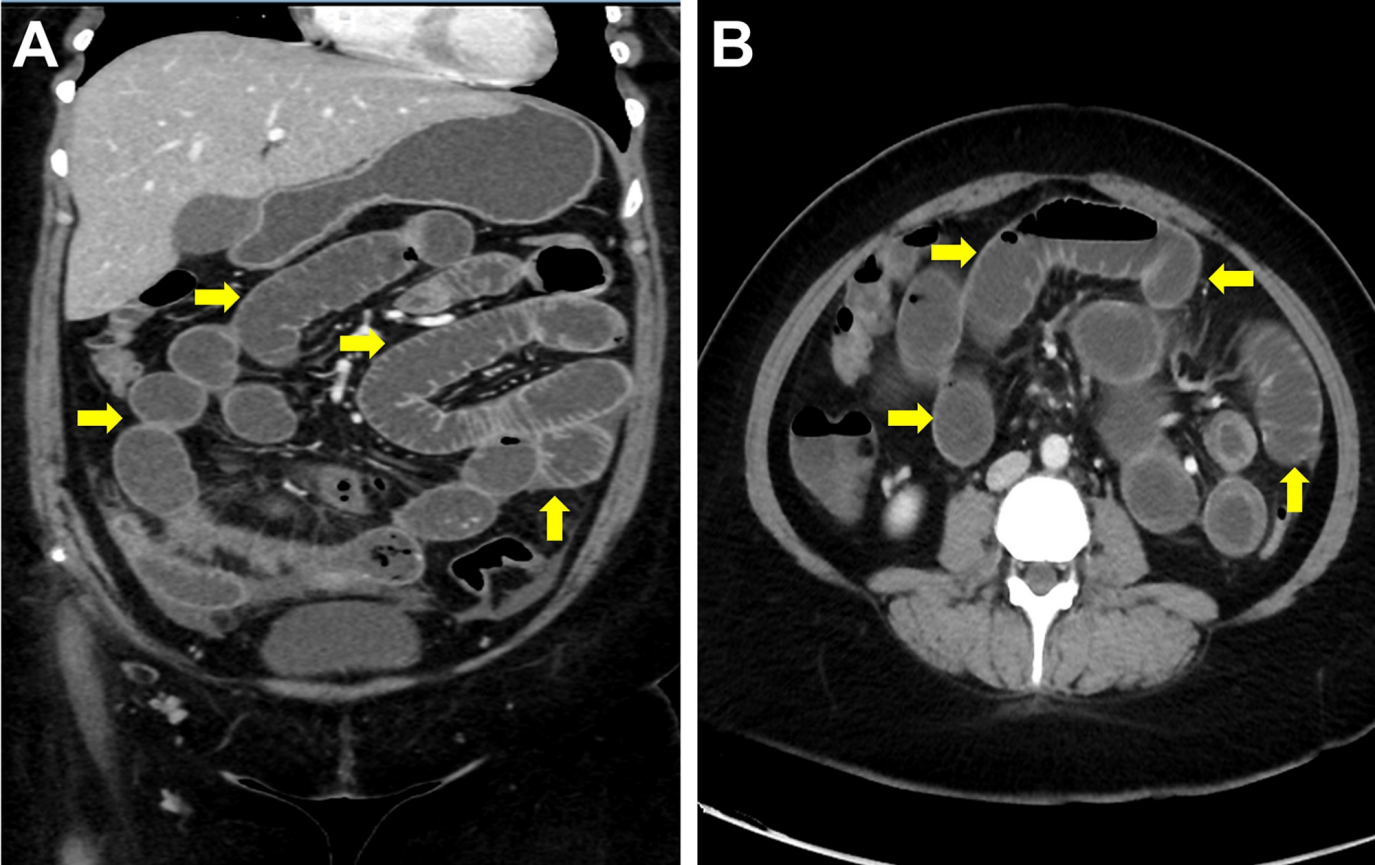
Coronal (A) and cross-sectional (B) contrast-enhanced computed tomography scans showing distended small bowel with a thickened edematous intestinal wall (arrows).

The patient underwent an abdominal x-ray which showed multiple air-fluid levels (Figure 1). Contrast-enhanced computed tomography scan revealed a soft tissue lesion involving the right side of the mesentery measuring about 6.2 x 3.2 x 1.8 cm and exhibiting surrounding desmoplastic reaction and tethering of the adjacent structures. It was encasing and infiltrating the adjacent ileocolic region and resulting in a mildly distended small bowel with thickened edematous intestinal wall and adjacent fat stranding (Figure 2). There were multiple lymphadenopathies. Differential diagnoses included carcinoid tumors and inflammatory bowel disease.

**Figure 3:**
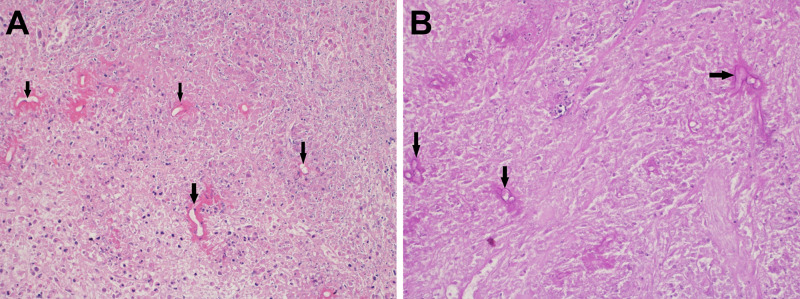
Microscopic examination of the resected mesenteric mass. (A) Hematoxylin and eosin staining revealed fungal hyphae within necrotizing granuloma and surrounded by intense eosinophilic material, most likely representing the Splendore-Hoeppli phenomenon (arrows). (B) Periodic Acid Schiff staining showing numerous broad and thin-walled fungal hyphae (arrows).

In view of potential small bowel obstruction and a malignant mass, the patient underwent exploratory laparotomy. The right-sided mesenteric mass was identified and resected. Hematoxylin and eosin staining revealed fungal hyphae within necrotizing granuloma and surrounded by intense eosinophilic material, most likely representing the Splendore-Hoeppli phenomenon (Figure 3A). Periodic Acid Schiff staining revealed numerous broad and thin-walled fungal hyphae (Figure 3B). The background revealed marked necrosis and a mixed inflammatory infiltrate rich in eosinophils. The final histopathologic diagnosis was consistent with gastrointestinal basidiobolomycosis. Polymerase chain reaction (PCR) of panfungal specific primers confirmed the presence of *B. ranarum* pathogen.

The patient had an uneventful postoperative course. The patient received itraconazole therapy. At six weeks follow-up, the patient was symptom-free. The plan was to continue therapy for additional 12 weeks and assess the patient again in the clinic with the radiographic examination. 

## Discussion

Basidiobolomycosis is a rare fungal infection [1]. It is largely a subcutaneous infection and its gastrointestinal involvement is an uncommon phenomenon [4,5]. It is mostly reported in countries with very arid climates, particularly Saudi Arabia and other Middle Eastern countries (for example, Iran) and the United States (specifically, Arizona State) [1,5]. The infection largely exhibits gender and age propensity for males and children, respectively [1,5]. It displays a tendency to infect immunocompetent individuals and no risk factors are clearly delineated [6]. Our patient was a female immunocompetent adult.

The precise mechanism of *B. ranarum* transmission is not certainly defined. Nonetheless, with regard to subcutaneous disease, it is most likely contracted following an insect bite or minor skin trauma. Conversely, with regard to gastrointestinal disease, it is most likely contracted secondary to ingestion of objects polluted by *B. ranarum*, such as foodstuff, animal feces, and outdoor soil. The latter mechanism appropriately rationalizes the high proportion of gastrointestinal basidiobolomycosis cases, particularly among children [5,7]. 

With regard to blood tests, elevated erythrocyte sedimentation rate, eosinophilia, and neutrophilic leukocytosis are frequent laboratory findings recognized in patients with gastrointestinal basidiobolomycosis [1,5,6]. All the abovementioned laboratory tests were found in our patient.

Anatomically, gastrointestinal basidiobolomycosis most frequently targets the colon. Signs and symptoms of gastrointestinal basidiobolomycosis are largely non-specific during clinical presentation [3]. Indeed, the lack of specific symptomatology is a major contributing factor to the delayed diagnosis of gastrointestinal basidiobolomycosis [3]. Nonetheless, in descending order, abdominal pain (90%), fever (40%), and abdominal mass (30%) are the most frequently reported complaints. Weight loss, constipation, and diarrhea are additional reported symptoms by patients [1,5]. Our patient had abdominal pain as the major presenting symptom.

Various radiographic modalities are utilized to characterize gastrointestinal basidiobolomycosis. Such radiographic modalities commonly abdominal x-ray, computed tomography, and upper gastrointestinal series. The most frequently recorded findings comprise abdominal masses of the colon, with an inflammatory reaction, bowel wall thickening, and abscess formation [8]. Differential diagnoses comprise inflammatory bowel disease, chronic tuberculosis, and neoplastic mass-occupying lesions, such as lymphoma.

Diagnosis is difficult to be made preoperatively. Several case reports employed preoperative diagnostic procedures, such as endoscopy and biopsy, however, they largely failed to establish the diagnosis [6]. Similar to previous cases, fungal culture was not performed as it was not contemplated preoperatively. It has been depicted that positive culture of *B. ranarum* is attained in 50% to 65% of the cases [5,6]. Hence, a negative culture does not firmly dismiss the diagnosis. Molecular diagnostics employing PCR assays with high sensitivity and specificity have been reported. However, not all healthcare institutions possess this technology [9].

Pathologic examination of the resected gastrointestinal mass establishes the definitive diagnosis. Distinctively, gastrointestinal basidiobolomycosis is manifested by the occurrence of Splendore-Hoeppli bodies, enrichment of eosinophils, and presence of abundantly radiating eosinophilic granular materials [10].

Surgical excision plus antifungal therapy represents the standard of care in the management of gastrointestinal basidiobolomycosis [1,5,6]. This approach aims to extensively eradicate the focus of infection and prevent relapse. There is no universally agreed-upon statement regarding the best antifungal agent. Azoles are often effective, particularly prolonged itraconazole treatment. Other azoles -- including voriconazole, posaconazole, fluconazole, and ketoconazole -- are less commonly utilized and have been reported by some scattered case reports [10,11]. Conversely, *B. ranarum* often exhibits resistance to amphotericin B in close to 50% of the cases [12].

The prompt diagnosis followed by treatment is key to guarantee a favorable prognosis. This is because delayed management can culminate in disseminated disease with serious consequences, such as mortality up to 16-20% of cases if left untreated [1,4]. Additionally, postponed treatment can result in unwanted complications, for example, mass-occupying obstructive uropathy, colon perforation, duodenobiliary fistula, and esophageal varices [13].

## Conclusions

Gastrointestinal basidiobolomycosis is an uncommon fungal infection. Its diagnosis is challenging owing to the natural rare incidence and presence of non-specific symptomatology that may mimic other common conditions. Pathologic examination findings are distinctive and can establish the definitive diagnosis, in conjunction with the relevant clinical history and blood test results. Prompt management with surgery and antifungal therapy is key to mitigate morbidity and mortality. Lastly, although infrequent, however, gastrointestinal basidiobolomycosis should be contemplated in patients presenting with abdominal pain, hematologic eosinophilia, and inflammatory gastrointestinal mass.
